# Homozygosity Mapping and Targeted Sanger Sequencing Identifies Three Novel *CRB1* (Crumbs homologue 1) Mutations in Iranian Retinal Degeneration Families

**DOI:** 10.18869/acadpub.ibj.21.5.294

**Published:** 2017-09

**Authors:** Mohammad Ghofrani, Mahin Yahyaei, Han G. Brunner, Frans P.M. Cremers, Morteza Movasat, Muhammad Imran Khan, Mohammad Keramatipour

**Affiliations:** 1 Department of Medical Genetics, Tehran University of Medical Sciences, Tehran, Iran; 2 Department of Human Genetics, Radboud University Medical Center, Nijmegen, the Netherlands; 3 Donders Institute for Brain, Cognition and Behaviour, Radboud University Medical Center, Nijmegen, the Netherlands; 4 Eye Research Center, Tehran University of Medical Sciences, Farabi Eye Hospital, Tehran, Iran

**Keywords:** Retinal degeneration, Retinitis pigmentosa, Leber congenital amaurosis, Mutation, Iran

## Abstract

**Background:**

Inherited retinal diseases (IRDs) are a group of genetic disorders with high degrees of clinical, genetic and allelic heterogeneity. IRDs generally show progressive retinal cell death resulting in gradual vision loss. IRDs constitute a broad spectrum of disorders including retinitis pigmentosa and Leber congenital amaurosis. In this study, we performed genotyping studies to identify the underlying mutations in three Iranian families.

**Methods:**

Having employed homozygosity mapping and Sanger sequencing, we identified the underlying mutations in the crumbs homologue 1 gene. The CRB1 protein is a part of a macromolecular complex with a vital role in retinal cell polarity, morphogenesis, and maintenance.

**Results:**

We identified a novel homozygous variant (c.1053_1061del; p.Gly352_Cys354del) in one family, a combination of a novel (c.2086T>C; p.Cys696Arg) and a known variant (c.2234C>T, p.Thr745Met) in another family and a homozygous novel variant (c.3090T>A; p.Asn1030Lys) in a third family.

**Conclusion:**

This study shows that mutations in *CRB1* are relatively common in Iranian non-syndromic IRD patients.

## INTRODUCTION

Inherited retinal diseases (IRDs) are a group of genetic disorders characterized by progressive degeneration of retinal cells and vision loss[1]. These disorders affect approximately 1 in 4,000 people, i.e. more than 2.4 million people are affected worldwide[2]. IRDs are well-known for their genetic and clinical heterogeneity as mutations in 240 genes are shown to be involved (https://sph.uth.edu/retnet/) and affected people represent different phenotypes in terms of onset, progression, and severity of the disease[3]. IRDs can be subdivided into non-syndromic forms such as retinitis pigmentosa (RP) and Leber congenital amaurosis (LCA), as well as syndromic forms such as Bardet-Biedl and Usher syndromes[1].

LCA is the most severe and congenital form of IRDs; the prevalence of which varies from 1:30,000 to 1:81,000. Clinical characteristics of the disease are severe visual impairment at birth, most of the time a non-detectable electroretinogram, sluggish or absent pupillary response, and oculodigital reflexes. Some patients also have hyperopia and nystagmus[4].

Generally, RP is less severe compared to LCA and has a later age of onset[4]. The earliest characteristic of the disease is night blindness, followed by peripheral vision loss and tunnel vision, and some patients will become legally blind[5]. Prevalence of the disease is estimated to be approximately 1 in 4,000 worldwide[6]. Bone spicule pigmentation, attenuation of retinal vessels, a waxy pallor appearance of optic disc, and various levels of retinal atrophy can be seen in the fundus of RP patients. Electroretinography of RP patients show severe reduction in a and b waves[6]. RP can be inherited as an autosomal dominant (adRP, about 30%-40% of cases), autosomal recessive (arRP, 50%-60%, with sporadic cases responsible for about 45% of all RP patients), or X-linked (xlRP, 5%-20%). Digenic inheritance has also been reported rarely.

A recessive mode of inheritance is more prevalent in countries like Iran due to the high prevalence of consanguineous marriages. Homozygosity mapping has been proven to be very successful in the identification of causal genes in such cases. This method involves detection of the disease locus in DNA of affected children of a consanguineous family by the fact that affected siblings carry identical homozygous chromosomal segments in which the causal variant is suspected. Homozygous regions can be identified using large sets of single nucleotide polymorphism (SNP) markers[7,8]. The advent of dense SNP-based genotyping microarrays has made homozygosity mapping faster and more effective[3]. Homozygosity mapping is particularly useful for mutation detection in genetically heterogeneous conditions, like IRDs, since it can minimize the number of genes that need to be sequenced to find the causative mutation[9].

Despite the genetic heterogeneous nature of the disease, some of these genes are mutated more often in certain groups of IRD. One of these frequently mutated genes is crumbs homologue 1 (*CRB1*; OMIM#604210), which is a human homologue of a Drosophila melanogaster gene that encodes crumbs protein. Mutations in *CRB1* are responsible for 10-15% of LCA cases and 4% of all cases of RP[10,11], and cumulatively they cause visual impairment in an estimated 80,000 patients worldwide[12]. The majority of the mutations in this gene are in exon 9 (41% of the cases) and exon 7 (27% of all cases)[13,14].

Previous studies have shown that up to 6.5% of arRP patients have a mutation in *CRB1*, which is located at chromosome 1q31.3 and consists of 12 exons[14]. This gene is expressed in the brain and retina, and an alternative splicing at 3’ end of the gene leads to the synthesis of a longer protein with 1406 amino acids and a shorter one with 1376 amino acids[15]. Both of these proteins have 19 epidermal growth factor (EGF)-like domains, three laminin A globular-like domains and a signal peptide. In addition, the longer isoform has a single transmembrane and a small 37-amino-acid intracellular domain[16]. CRB1 is located in the subapical region of the photoreceptor, and its intracellular domain creates complexes with intracellular proteins, which altogether are called the crumbs protein complex[17]. These protein complexes take part in the organization of macromolecular complexes crucial for cell polarity, morphogenesis, and maintenance of retina[16]. Various studies have shown that mutations in *CRB1* can cause different retinal dystrophies ranging from LCA to RP[13,14].

Upon funduscopic analysis of patients with IRDs caused by mutations in *CRB1* gene, namely LCA type 8 (LCA8; 613835) and RP type 12 (RP12; 600105), additional specific features may be found, including preservation of para-arteriolar retinal pigment epithelium (PPRPE) and coats-like vasculopathy. PPRPE is the preservation of RPE cells adjacent to and under retinal arterioles, while all other RPE cells of retina have degenerated. Coats-like vasculopathy increases the permeability of retinal vessels resulting in exudative retinal detachment and often is accompanied by massive deposits of subretinal lipids[12].

Very little is known about the genetic landscape of IRDs in the Iranian population. In this study, we searched for genetic defects in Iranian IRD families using homozygosity mapping and Sanger sequencing and identified three families with *CRB1* variants.

## MATERIALS AND METHODS

### Subjects

In total, 86 autosomal recessive, four autosomal dominant, and three X-linked pedigrees with a minimum of two members showing the signs and symptoms of retinal degeneration from various geographical parts of Iran (Iranian IRD cohort) were included in this study. A written informed consent adhering to the tenets of the Declaration of Helsinki was obtained from the probands of each family. Peripheral blood samples of all pedigree members were collected and mixed with EDTA anticoagulant (Merck KGaA, Darmstadt, Germany). A phenol-chloroform method was used to extract DNAs from peripheral leukocytes[18], and DNAs were stored at 4°C until further analysis.

Two affected individuals from 10 selected pedigrees were tested by affymetrix CytoScan HD™ whole genome SNPs array. Homozygous regions were determined by an online tool, i.e. Homozygosity Mapper (http://www.homozygositymapper.org)[19]. Homozygous regions of more than 2 Mb were ranked according to their size. Previous studies have indicated that the largest homozygous segments are more likely to carry the causative mutation[20]. Genes associated with retinal disease phenotypes residing in these regions were prioritized for Sanger sequencing. Sanger sequencing was also used for segregation analysis.

### Clinical characterization

Diagnoses of the patients were established by a trained ophthalmologist using Electroretinography (Metrovision MonPack3, 4 rue des Platanes, 59840 Pérenchies, France), evaluation of visual acuity, and Funduscopy (Topcon TRC-50EX Retinal Camera, 75-1. Hasunuma-cho, Itabashi-ku, Tokyo, Japan).

### Genotyping

Primers used for PCR reactions (Table 1) were designed by Primer3 software. PCR products were purified with exonuclease and thermosensitive alkaline phosphatase (Thermo Fisher scientific™, Waltham, Massachusetts, USA) and analyzed by Sanger sequencing using BigDye® Terminator v3.1 Cycle Sequencing Kit (Applied Biosystems™, Foster city, California, USA) ABI 3730XL platform (Applied Biosystems™). To assess the pathogenicity of the missense variants identified in this study, various software were used, including SIFT (http://sift.jcvi.org), PolyPhen-2 (http://genetics.bwh.harvard.edu/pph2), and Combined Annotation Dependent Depletion (CADD) score assessment (http://cadd.gs.washington.edu/score). The frequency of these variants was also determined using Exome Aggregation Consortium (ExAC), Cambridge, MA, USA (http://exac.broadinstitute.org; accessed on 2015/11/26).

**Table 1:** List of primers and sequences

Exon #	Forward primer (5’-3’)	Reverse primer (5’- 3’)
1	CGCTCCTCTCTGAGACAGAC	TTTTATAGAACATGCAACATTATCC
2.1	AATGAGTTTGGTTGAGGCAG	ATATCCAGCAGGGCAGATG
2.2	CAGTGGGACAATCTGTGAAAC	AATGTCACCTCTGCTTCTGC
3	GCTAAATTATGAACACTTTGCTAAAAC	GGTAAAATAGTTCATGGTCAGGG
4	CATGGGTCTTGGGTTGATAG	TTCATTTCATTTGCTATAAGCG
5	AACCTCCTTTTAGGCAAATG	GGTTAAAGCCATGGTCTGC
6.1	GAGCTATTCATGCACTTCTGC	GCCTCTGCAAATATTACCTCC
6.2	GAAGCTGGAGCTGCTAAGTG	TTTGCTGTTTCTGCTCTGC
7.1	TCCATCCCTTCTGTCTTTTG	TCCTAGGTTTTGTGAAGACTGA
7.2	TGGTGGGTCAGTAACATCATC	GCAATGCTGACTCCAAACTC
8	CAGATATGTGGTTTCACCGTC	TCTGTGTTTGCTCTTGGAAC
9.1	AAAAGCAACTAGCACAGTATGTAAC	AACTGCAAACAGCCAGTGAC
9.2	TGTGGGAGACAGAGCTATTGA	CTTGAGGAGAGAGCTTTCCAA
10	CTTTTCTTGAATGAGATGAACAAG	GAACTTTGAGTAATCCCATCATTC
11	GCTGTTCCAGAGAGATAAGGC	CTCAACAACTGGCTCGTCAT
12	TTCCTGAGTAGTTCCATTGTCC	CCCAGTTGCAGATTAACATTG

List of primer used for Sanger sequencing of *CRB1* gene. Multiple overlapping primers were used to amplify and sequence exons 2, 6, 7, and 9.

## RESULTS

**Fig. 1:**
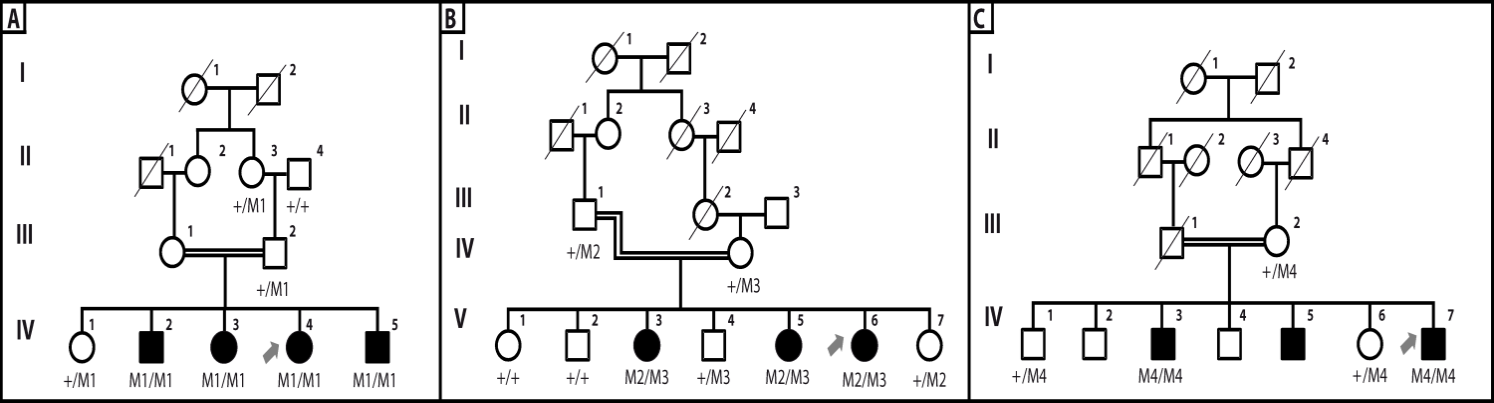
Pedigrees and segregation of CRB1 variants of the Iranian families participated in this study. (A) Family W13-0007; (B) family W13-1504; (C) family W13-1493. Affected individuals are indicated with filled symbols, whereas unaffected relatives are shown by open circles or rectangles. Symbols with a slash depict deceased individuals. Arrows indicate probands. +, wild type allele; M1, c.1053_1061del (p.Gly352_Cys354del), M2, c.2234C>T (p.Thr745Met); M3, c.2086T>C (p.Cys696Arg); M4, c.3090T>A (p.Asn1030Lys). Roman letters represent number of generations.

**Fig. 2:**
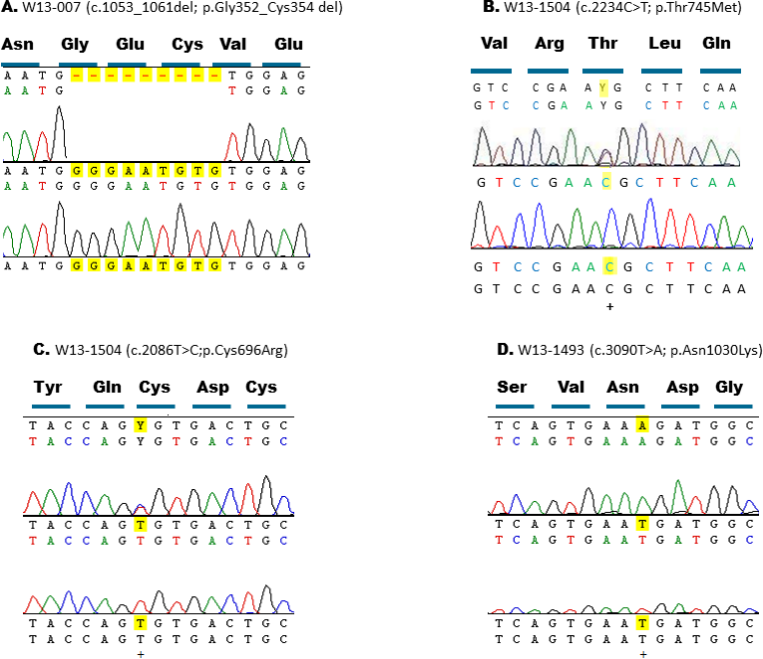
Sequence chromatograms of the CRB1 variants. (A) The novel homozygous mutation (c.1053_1061del; p.Gly352_Cys354 del) in IV:4 of family W13-0007; (B) the recurrent mutation (c.2234C>T; p.Thr745Met) in V:6 of family W13-1504; (C) the novel mutation (c.2086T>C; p.Cys696Arg) in V:6 of family W13-1504; (D) the novel mutation (c.3090T>A; p.Asn1030Lys) in IV:7 of family W13-1493. Letters highlighted in yellow indicate the altered nucleic acid.

Employing homozygosity mapping, one family (W13-0007) out of the 10 families analyzed by whole genome SNP array, showed linkage to the chromosomal region containing *CRB1*. This family is a consanguineous family from Mazandaran province in the north of Iran, with four affected and one unaffected siblings. The parents are first cousins (Fig. 1A). A total of 63 Mb of the genome was shared between two affected members of the family, who were analyzed by Affymetrix CytoScan HD array (W13-0007; IV:3 and W13-0007; IV:4). The largest homozygous region was about 42 Mb and contained three IRD-associated genes: *CRB1*, *RD3* (retinal degeneration 3; OMIM# 180040), and *USH2A* (Usher syndrome type 2A; OMIM# 608400). Bearing in mind the contribution of the disease genes towards IRD phenotypes, *CRB1* was prioritized for sequencing. Sanger sequencing of *CRB1* identified a novel 9-bp in-frame homozygous deletion (Fig. 2A) in exon 5 (c.1053_1061del; p.Gly352_ Cys354del), which was segregating in the family (Fig. 1A).

Since the majority (68%) of *CRB1* mutations reside in exons 7 and 9, these exons in our Iranian IRD panel were sequenced. In this way, we identified two mutations, a previously identified heterozygous variant (c.2234C>T; p.Thr745Met) https://databases.lovd.nl/shared/variants/CRB1/unique[22] in exon 7 (Fig. 2B) and a novel homozygous variation (c.3090T>A; p.Asn1030Lys) in exon 9 (Fig. 2D), in probands of families W13-1504 (Fig. 1B) and W13-1493 (Fig. 1C), respectively. Subsequently, all other exons of *CRB1* were sequenced in the proband of family W13-1504, and a second likely pathogenic variant (c.2086T>C; p.Cys696Arg) was found in exon 6 (Fig. 2C). Family W13-1504 was from Eastern Azerbaijan, and family W13-1493 was from the Zanjan province. The identified variants are segregated with the disease in the respective families.

**Fig. 3:**
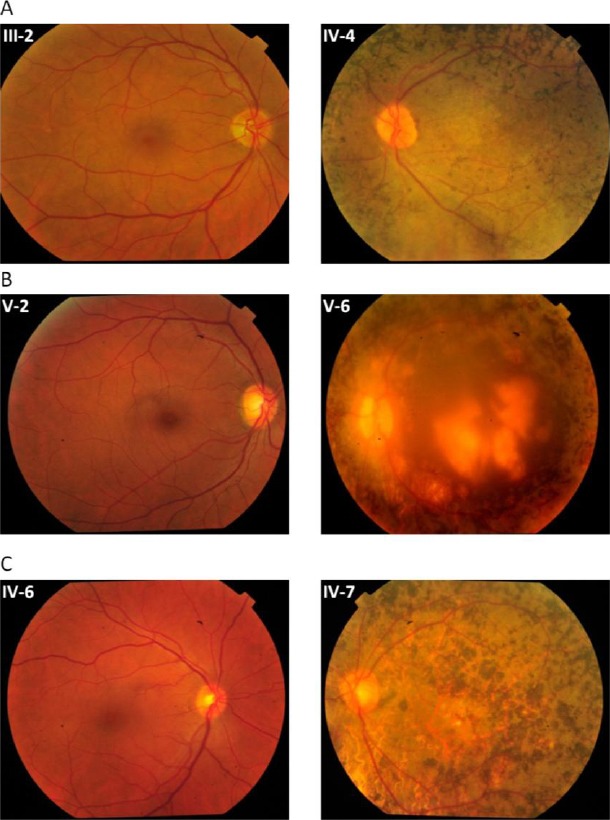
Fundus photographs of the selected normal and affected individuals. (A) W13-0007, individuals III-2 (normal) and IV:4 (affected); (B) W13-1504 individuals V-2 (normal) and V:6 (affected); (C) W13-1493 individuals IV-6 (normal) and IV:7 (affected)

Evaluation of family histories showed that the first symptom of RD appeared in all affected members of all three families in their childhood. The speed of disease progression varies among different families and even among affected siblings of one family. Upon funduscopic examination, typical features of RP were observed in all three families and included peripheral bone spicule pigmentation, retinal blood vessels attenuation, pallor optic disk, and maculopathy (Fig. 3). In addition, all members of family W13-1504 had nystagmus and bilateral cataract. Clinical characteristics of the patients are summarized in Table 2.

**Table 2:** Clinical characteristics of the affected pedigree members participating in this study

Patient	Current age (year)	Gender	Age of onset (year)	Visual acuity	Fundus appearance
W13-0007: IV-3	34	F	6	Hand motion	Peripheral bone spicules, attenuated retinal blood vessels, macular degeneration
W13-0007: IV-4	32	F	8	OD: 5/10 OS: 5.5/10	Peripheral bone spicules, attenuated retinal blood vessels, macular degeneration
W13-1504: V-3	49	F	3	LP+	Peripheral bone spicules, attenuated retinal blood vessels, macular coloboma, nystagmus, cataract
W13-1504: V-6	39	F	3	LP+	Peripheral bone spicules, attenuated retinal blood vessels, macular coloboma, nystagmus, cataract
W13-1493: IV-3	60	M	4	TB	Not applicable because of severe cataract
W13-1493: IV-7	43	M	4	LP+	Peripheral bone spicules, attenuated retinal blood vessels, macular degeneration

F, female; M, male; TB, total blindness; LP+, light perception; OD, *oculus dexter*, right eye; OS, *oculus sinister*, left eye.

All of the missense variants found in this study were analyzed by predictive tools. The result of these analyses, generally, support the pathogeniecity of the variants (Table 3).

**Table 3:** *In-silico* analysis of the missense mutations identified in this study

Family ID	DNA variant; Chromosomal position	Protein change	Grantham	PhyloP	SIFT	PolyPhen-2	CADD score	ExAC Minor allele frequency	Ref.
W13-1504	c.2086T>C; Chr1-197391044T>C	p.Cys696Arg	180	4.73	Del	PD	24	Absent	This study
W13-1504	c.2234C>T; Chr1-197396689C>T	p.Thr745Met	81	4.16	Del	PD	23.6	T=0.000083	[22]
W13-1493	c.3090T>A; Chr1-197404083T>A	p.Asn1030Lys	94	-0.36	Del	PD	18.17	Absent	This study

SIFT, sorting intolerant from tolerant; PolyPhen-2, polymorphism phenotyping; CADD, combined annotation dependent depletion; ExAC, Exome Aggregation Consortium; Del, deleterious; PD, probably damaging

## DISCUSSION

We identified the underlying mutations in the *CRB1* gene in three Iranian retinal degeneration families. Although consanguineous marriages are common in Iranian society[23], which makes the Iranian gene pool a valuable asset for genetic studies, very little is known about genetics of retinal degeneration in the Iranian population. To our knowledge, only two studies have been reported on genetic causes of Iranian IRD patients[24,25].

Interestingly, a combination of a known mutation in exon 7 and a novel mutation in exon 6 of *CRB1* was found in affected members of the consanguineous family W13-1504. This result is not unprecedented as compound heterozygous variants that have previously been reported in other consanguineous families with recessive diseases, inside and outside Iran[26,27] and even in *CRB1* in non-Iranian RP patients[28].

**Fig. 4:**
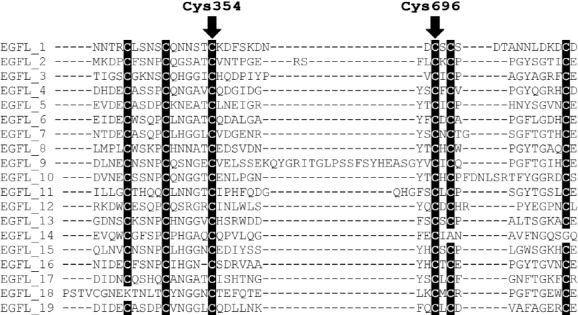
Conservation of cysteine residues in epidermal growth factor (EGF) domains of human CRB1 (UniProtKB - P82279). The mutated cysteine residues are present in all 19 EGF-like domains

**Fig. 5:**
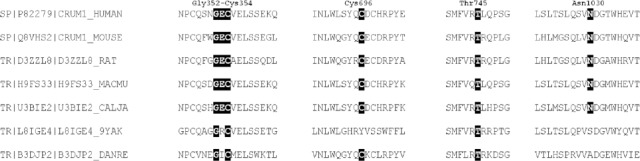
Conservation of mutated residues identified in this study in full-length CRB1 from different species. Amino acids are depicted with one letter code. Black boxes show the conserved amino acid residues in different species.

Two of three novel mutations found in this study involve cysteine residues. Cysteine is crucial in disulfide bonds formation and subsequently in three dimensional structure of CRB1[13,14]. The mutation found in family W13-0007 deletes a segment of 3 amino acids (p.Gly352_Cys354 del) from CRB1. This segment is a part of the 9^th^ EGF-like domain in CRB1. Cysteine 354 forms a disulfide bond with another cysteine at position 343. Patients of family W13-1504 have compound heterozygous mutations. One of the mutations (c.2234C>T, p.Thr745Met) has previously been reported and is among the most prevalent *CRB1* variants (https://databases.lovd.nl/shared/variants/CRB1/unique)[22]. However, the other one (c.2086T>C, p.Cys696Arg) is a novel variant in exon 6, which leads to the replacement of a cysteine residue at position 696 by an arginine. Cysteine at position 696 is located in 12^th^ EGF-like domain and makes a disulfide bond with cysteine 681. None of these two changes are among highly conserved cysteine residues of CRB1 protein reported previously[13]. However, when we aligned all 19 EGF-like domains of human CRB1 protein, it became evident that both of the mutated cysteine residues are conserved inside these domains (Fig. 4). Comparing the full-length protein sequence of CRB1 among seven species also showed that cysteine residues at positions 354 and 696 were conserved during the evolution of these species (Fig. 5). Conservation of these cysteine residues inside different EGF-like domains and among CRB1 protein of different species demonstrates their importance in structure and/or function of human CRB1 protein.

The third novel homozygous variant (c.3090T>A; p.Asn1030Lys) found in family W13-1493 is considered causal as SIFT and PolyPhen2 predicted it to be deleterious and disease causing, it is segregating in the family, and also the variant is absent in all databases containing sequence variants in control individuals.

Considering that IRDs are genetically heterogeneous diseases, and none of the genes involved have a high frequency, results of this study show that mutations in *CRB1* are relatively common in Iranian IRD patients. Identification of pathogenic events at the molecular level can help clinicians and health professionals improve and expedite the diagnosis of the disease and provide patients and their families with a more accurate genetic counseling. Increasing the knowledge about molecular mechanisms involved in IRD pathogenesis may also lead to the development of new treatment options for affected individuals in the future.
